# How Gut Bacterial Dysbiosis Can Promote *Candida albicans* Overgrowth during Colonic Inflammation

**DOI:** 10.3390/microorganisms10051014

**Published:** 2022-05-12

**Authors:** Samir Jawhara

**Affiliations:** 1 UMR 8576—UGSF—Unité de Glycobiologie Structurale et Fonctionnelle, Centre National de la Recherche Scientifique, Institut National de la Santé et de la Recherche Médicale U1285, Université Lille, F-59000 Lille, France; samir.jawhara@univ-lille.fr; Tel.: +33-(0)3-20-62-35-46; Fax: +33-(0)3-20-62-34-16; 2 Medicine Faculty, University of Lille, F-59000 Lille, France

**Keywords:** *Candida albicans*, β-glucans, chitin, dysbiosis, microbiota, Crohn’s disease, DSS, MBL, TLR

## Abstract

*Candida albicans* is a commensal opportunistic yeast, which is capable of colonising many segments of the human digestive tract. Excessive *C. albicans* overgrowth in the gut is associated with multiple risk factors such as immunosuppression, antibiotic treatment associated with changes to the gut microbiota and digestive mucosa that support *C. albicans* translocation across the digestive intestinal barrier and haematogenous dissemination, leading to invasive fungal infections. The *C. albicans* cell wall contains mannoproteins, β-glucans, and chitin, which are known to trigger a wide range of host cell activities and to circulate in the blood during fungal infection. This review describes the role of *C. albicans* in colonic inflammation and how various receptors are involved in the immune defence against *C. albicans* with a special focus on the role of mannose-binding lectin (MBL) and TLRs in intestinal homeostasis and *C. albicans* sensing. This review highlights gut microbiota dysbiosis during colonic inflammation in a dextran sulphate sodium (DSS)-induced colitis murine model and the effect of fungal glycan fractions, in particular β-glucans and chitin, on the modification of the gut microbiota, as well as how these glycans modulate the immuno-inflammatory response of the host.

## 1. Introduction

**Figure 1:**
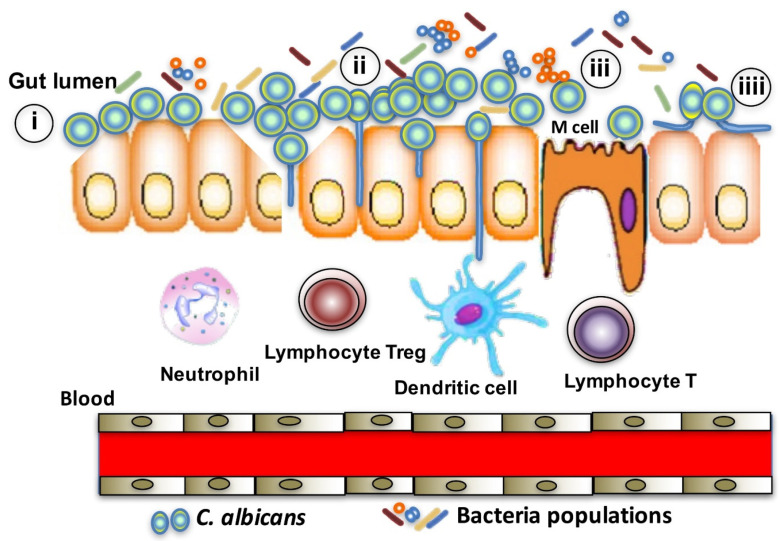
Schematic representation of some mechanisms that promote the passage of *C. albicans* through the intestinal barrier. (i) Dysbiosis, associated or not with inflammatory lesions of the intestinal mucosa and effraction of the intestinal barrier; (ii) a decrease in watertightness of the epithelium (tight junctions) and mucus layer; (iii) endocytosis by M cells (microfold cells) situated in the intestinal epithelium, dendritic cells or macrophages; (iiii) endocytosis by epithelial cells, such as the interaction between Als3 or Ssa1 on the hyphal surface of *C. albicans* and epithelial cells of E-cadherin.

*Candida albicans* is a commensal yeast and a natural saprophyte of the human digestive tract and vagina [1,2]. The digestive tract is considered to be the main reservoir for infection [3,4]. It is known that candidaemia and disseminated candidosis are usually endogenous and mainly originate from the gut microbiota [4]. Excessive colonisation of the digestive mucosa by *C. albicans* is associated with risk factors such as immunodeficiency and changes to the digestive tight junction, intestinal barrier and gut microbiota dysbiosis following treatment with antibiotics, radiotherapy or immunotherapy (Figure 1) [5]. These factors favour translocation of the yeast across the epithelial barrier and possible haematogenous dissemination resulting in serious disseminated infections [2]. *C. albicans* possesses a cell wall rich in glycans. This cell wall is the first contact between the yeast and its host and plays an important role in modulation of the immune response of the host [6]. Interaction between Candida and host cells is critical for initial fungal colonisation of the host and for induction of different processes leading to infection [4].

The cell wall of *C. albicans* is a complex layered and dynamic structure [7]. The structure of the *C. albicans* cell wall consists of deep layers of chitin and more or less dense β-1,3 and β-1,6 glucans in the intermediate layers [8]. Chitin is a β(1,4)-linked homopolymer of N-acetylglucosamine that folds in an anti-parallel manner forming intra-chain hydrogen bonds [9]. These chitin microfibril chains are covalently attached to β(1,3)-glucan to form the inner skeleton of the *C. albicans* cell wall [9]. The surface of the cell wall is covered with phosphopeptidomannan (PPM), which is not linked covalently [10]. *C. albicans* is capable of synthesising β-mannosyls, which are associated with α-mannosyls in PPM, while they are expressed electively and associated non-covalently at the cell wall surface in a secreted glycolipid named phospholipomannan (PLM) [10]. In his review of the *C. albicans* cell wall, Poulain describes the link between *C. albicans* and Crohn’s disease (CD) [4]. 

Numerous *C. albicans* cell wall adhesins have been shown to exert an influence on epithelial attachment, including Als3 (a member of the *C. albicans* agglutinin-like sequence) and Ssa1 (a member of the heat-shock protein family), which induce epithelial cell endocytosis of *C. albicans* hyphae by binding to the epithelial receptor E-cadherin [11,12]. Additionally, Als3 and Ssa1 are recognised by the epidermal growth factor receptor (EGFR/HER1) and HER2 that induce epithelial cell endocytosis of *C. albicans* (Figure 1) [13].

## 2. Role of *C. albicans* in Colonic Inflammation

The role of yeasts in modulation of colonic inflammation has received growing interest from researchers for two reasons. The first concerns the yeast *C. albicans*, which is both a frequent commensal of the digestive tract and an opportunistic pathogen [3]. The second concerns a possible link between *C. albicans* and CD [4,14,15]. Different clinical and experimental studies have revealed that infection with *C. albicans* can generate a panel of anti-glycan antibodies, described under the acronyms ASCA (anti-*Saccharomyces cerevisiae* antibodies), ALCA (anti-laminaribioside) and ACCA (anti-chitobioside), which are serological markers of CD [6,14,16,17]. CD is a chronic transmural inflammatory bowel disease (IBD) that most commonly affects the distal ileum and colon but may occur in any part of the digestive tract [18].

The mechanisms responsible for the development of CD result from the interaction between genetic susceptibility, environmental factors, infectious factors and/or immunological mechanisms. Chronic intestinal inflammation in CD is probably related to changes in gut microbiota composition and dysbiosis of the gut fungal microbiota [19].

Li et al. showed that fungal richness and diversity were significantly elevated in the inflamed mucosa compared to the non-inflamed mucosa in CD patients [19]. From an experimental point of view, the involvement of *C. albicans* overgrowth in mucosal damage was explored in a murine model of dextran sulphate sodium (DSS) induced pre-inflammation of the colon [20]. In this DSS model, *C. albicans* exacerbates colonic inflammation induced by DSS in mice and conversely, DSS colitis promotes *C. albicans* overgrowth [20]. In parallel, *C. albicans* overgrowth in mice with DSS-induced colitis generated ASCA, suggesting that circulating *C. albicans* mannan can induce the production of these glycan antibodies during intestinal inflammation [3,20].

This DSS model, which promotes the overgrowth of *C. albicans*, was also used to investigate the role of the gut microbial environment and the beneficial effects of probiotics on colonisation by Candida spp. and inflammation [15]. *Saccharomyces boulardii*, a variety of *S. cerevisiae*, known as a non-pathogenic yeast, is recognised to have a beneficial probiotic effect when administered orally as a lyophilised preparation to treat antibiotic-associated diarrhoea, acute infectious gastroenteritis and *Clostridioides difficile* infection [21,22]. After repeated administration, *S. boulardii* reaches steady-state concentrations in the colon within 3 days and is completely eliminated from stools 2–5 days after discontinuation [23]. In the DSS-induced colitis model, the administration of *S. boulardii* decreased colonisation by *C. albicans* as well as colonic inflammation in mice, with a reduction in pro-inflammatory cytokine expression and a difference in expression of TLR2 [15]. This study revealed that oral administration of *S. boulardii* promotes the elimination of *C. albicans*, reduces mucosal injury mediated via TLR and modulates cytokine expression [15]. 

In addition, among the dozen strains of *S. cerevisiae* (industrial yeasts used in human and animal foodstuffs) tested, one *S. cerevisiae* strain was found to have probiotic properties and was able to reduce both *C. albicans* overgrowth and colonic inflammation in mice [24]. Surprisingly, some yeast strains closely related to *S. cerevisiae* caused mucosal injury and mouse mortality [24]. Subsequently, the cell wall components (mannan and β-glucans) of *S. cerevisiae* exhibiting probiotic properties were extracted in order to define the molecular basis for the beneficial or deleterious effects of these components on the host immune response [24]. These studies revealed that in contrast to mannan, extracts of β-glucans (derived from *S. cerevisiae* or even *C. albicans*), described classically as pro-inflammatory, had beneficial activities against colonic inflammation and colonisation by *C. albicans* [24]. The strain of *S. cerevisiae* chosen in this study was used in a clinical trial in patients with irritable bowel syndrome (IBS) [25]. Treatment with this yeast gave promising results in these IBS patients via a reduction abdominal pain/discomfort scores [25]. By contrast, in a recent study showing that colonisation with *S. cerevisiae* enhanced the metabolism of purine, leading to an increase in uric acid production, Sendid et al. showed no correlation between the level of uric acid and ASCA levels in a clinical study of a cohort of CD patients, indicating that *S. cerevisiae* is not involved in the increase in uric acid levels in patients with CD [26].

The contribution of β-Mans to the virulence of Candida spp., in particular *C. glabrata*, a species frequently isolated in human disease, was explored using the DSS-induced colitis model [27]. Like *C. albicans*, *C. glabrata* is a pathogenic yeast that causes severe infections in humans, including bloodstream and mucosal infections [28,29]. *C. glabrata* is a significant clinical problem in immunocompromised patients where it can disseminate from the gut to cause systemic candidosis [30]. Systemic *C. glabrata* infections are associated with a higher mortality than *C. albicans* infections [31]. In the DSS-induced colitis model, *C. glabrata* deficient in β-Mans was less virulent than the parental strain in terms of colonisation, mortality rate and clinical and histological scores for intestinal inflammation, suggesting that β-Mans play a crucial role in the processes of pathogenesis/virulence of *C. glabrata* during intestinal inflammation [27].

The *C. albicans* transition from blastoconidia to hyphal forms plays a crucial role in *C. albicans* pathogenesis and this transition affects the fungal cell wall composition and immune reactivity [32,33]. Different virulence factors are expressed by the *C. albicans* hyphal form, including the Sap family of secreted aspartyl proteases, cell surface adhesins like Als3, Hwp1 and Hyr1, the pore-forming toxin (candidalysin) and pH-regulated antigen 1 (Pra1) [32,33,34]. Witchley et al. showed that *C. albicans* strains lacking SAP6 or the transcription factor Ume6 exhibited high colonisation fitness in the mouse gut [35]. In mice with DSS-induced colitis, vaccination of mice with *C. albicans* Als3 protein (NDV-3A) did not affect *C. albicans* morphology or intestinal fungal burden, but NDV-3A decreased *C. albicans*-associated damage [36].

**Figure 2:**
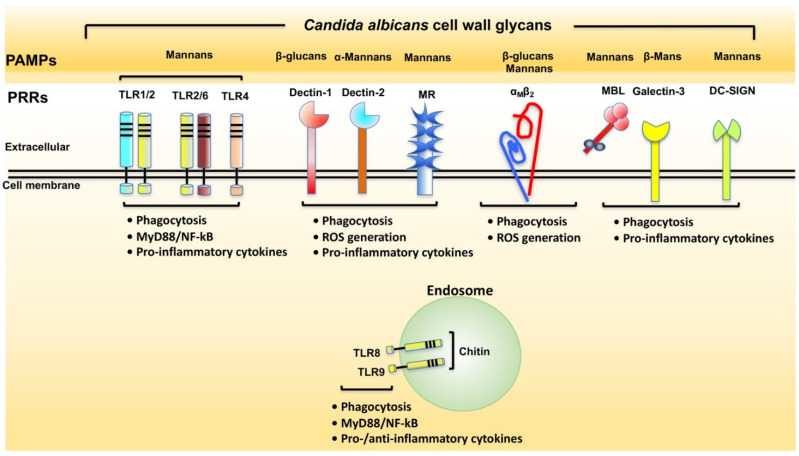
Diagrammatic representation of the main pattern recognition receptors (PRRs) involved in sensing *C. albicans* cell wall glycans.

Several classes of pattern recognition receptors (PRRs) have been implicated in the recognition of *C. albicans* pathogen-associated molecular patterns (PAMPs), including integrins, TLRs and C-type lectin receptors (CLRs), and excellent reviews have been published on this subject (Figure 2) [37,38,39]. These PRRs play a crucial role in the initiation of the innate immune response against pathogenic yeasts and in modulation of the inflammatory response [39]. With regards to integrins, α_M_β_2_ and α_X_β_2_ have been implicated in the recognition of the *C. albicans* cell wall [40,41,42]. Mice deficient in these integrins displayed increased susceptibility to systemic infection by *C. albicans* [40,41]. In terms of CLRs, *C. albicans* is recognised by several CLRs, including galectin-3, dectin-1 and mannose-binding lectin (MBL) [37]. It has been shown that galectin-3 recognises β-Mans of *C. albicans* [43]. In the DSS model, the potentialisation of inflammation induced by *C. albicans* was under the control of galectin-3 as well as TLR2 in mice, emphasising the role of these two receptors in the recognition of *C. albicans* [20]. In addition to galectin-3, dectin-1 recognises β-1,3-glucans of *C. albicans* [37,44]. Mice lacking dectin-1 are more susceptible to DSS-induced colitis than wild-type mice, indicating the result of altered responses to endogenous fungi [45].

## 3. Role of MBL and TLRs in Intestinal Homeostasis and *C. albicans* Sensing

**Figure 3:**
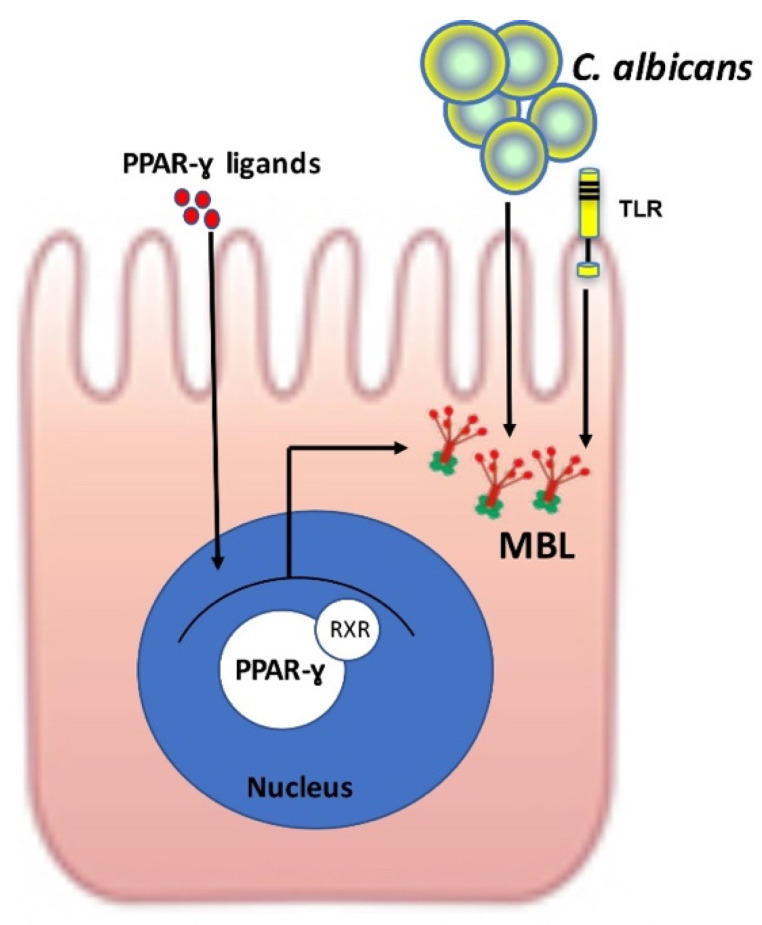
Expression of MBL in epithelial cells in response to *C. albicans* sensing and PPARγ activation. Epithelial cells produced MBL after *C. albicans* sensing alone or with PPARγ agonist pioglitazone treatment [56]. PPARγ and TLR sensing by *C. albicans* led to MBL production in the epithelial cells.

MBL is a soluble lectin present in serum [46]. It is mainly synthesized by hepatocytes before being released into blood circulation [46]. MBL is composed of a terminal amino region rich in cysteine followed by a collagenous region, a type C lectin (terminal carboxy domain) and what is known as the carbohydrate-recognition domain (CRD) at the C-terminal [47]. MBL induces activation of the lectin complement pathway after microorganism recognition through the CRD [48,49]. The MBL CRD senses polysaccharide patterns, such as D-mannose, N-acetylglucosamine and L-fucose, on different clinically relevant pathogens [50,51,52]. MBL circulates in the form of a hetero complex with MBL-associated serine protease (MASP) 1, 2 and 3 [53]. Only one form of human MBL has been characterised, while two forms of MBL (A and C) are found in rodents and monkeys [54]. Additionally, MBL-A has been considered to be the serum form while MBL-C has been called the liver form in rodents [55]. Choteau et al. reported that expression of MBL was observed in human intestinal epithelial cells (biopsies of colon from the operating room) and that this MBL expression was mediated by PPARγ and influenced by *C. albicans* sensing (Figure 3) [56]. In a murine model, the expression of two forms of MBL (A and C) was demonstrated in epithelial cells from the stomach, caecum and colon of C57BL/6 mice [56]. This expression was increased when the mice were colonised by *C. albicans* although serum levels did not vary [56]. A deficit of MBL favoured colonisation by *C. albicans* and dissemination of the yeast in the presence of DSS-induced colitis [56].

MBL can interact with other receptors of the innate immune system, such as TLR2 and TLR4 [57]. It has also been demonstrated that MBL reacts with TLR2 during infection with *Staphylococcus aureus* [58]. It is well known that TLRs are crucial innate immune components that recognise *C. albicans* PAMPs [59]. Mutations and dysregulation of TLRs are important factors that contribute to predisposition and susceptibility to IBD [60]. Different studies have shown that TLR activation by commensal bacteria contributes to colonic homeostasis and tolerance induction in the gut [61,62,63].

The role of TLR1, TLR2 and TLR6 has been explored in the DSS-induced colitis model [64]. TLR2, associated with TLR1 or TLR6, is involved in the recognition of *C. albicans* and maintenance of the intestinal barrier [64]. Additionally, TLR1 and TLR2 participate in the defence against colonisation/infection by *C. albicans*, with deficiency of these receptors leading to dissemination of the yeast. Conversely, TLR6 favours intestinal colonisation by *C. albicans* [64]. These experimental studies suggest that MBL and TLR1/TLR2/TLR6 regulate the expression of pro-inflammatory cytokines involved in Th1 and Th17 responses during colonic inflammation and play a crucial role in establishing a balanced immune response against *C. albicans* [56,64].

In addition to these experimental studies, a clinical study focused on the quantitative and qualitative variations in MBL in patients with CD and their possible link with the persistence of colonic inflammation. The study on 256 CD patients demonstrated that the MBL2 gene was associated with a quantitative deficit of MBL and a qualitative deficit of the MBL-MASP complex in healthy subjects and patients [65]. This polymorphism rs5030737 of the MBL2 gene was associated with high levels of ASCA in CD patients and was frequently associated with severe forms of the disease [65]. These experimental and clinical data demonstrate intestinal production of MBL for the first time, which is modulated by colonisation with *C. albicans*. They confirm the role of MBL and TLRs in intestinal homeostasis and defence against *C. albicans*. Furthermore, the clinical study on the group of CD patients demonstrated that a fault in the functional activity of the MBL-MASP complex in CD patients with polymorphisms of the genes for MBL2 and NOD2 could lead to a severe phenotype of the disease [65].

## 4. Impact of Colonic Inflammation on the Gut Microbiota and How a Decrease in Anaerobic Bacteria Populations Promote Fungal Overgrowth

The gut microbiota constitutes a natural barrier against the proliferation of opportunistic pathogens [66]. The current leaning is in favour of a predominant role of intestinal microbiota in the initiation and persistence of CD lesions. The role of the saprophytic gut microbiota as an initiator was strongly suspected from several studies conducted in animals that developed inflammatory colitis, where the presence of the gut microbiota was essential for the development of inflammation. Thus, mice (IL-10 knockout) developing inflammation of the intestinal mucosa under normal breeding conditions did not develop colitis under germ-free conditions [67,68]. Bacteria belonging to the phyla Bacteroidetes and Firmicutes dominate the gut and, to a lesser extent, species from Proteobacteria and Actinobacteria. Frank et al. demonstrated that patients with chronic IBDs had a decrease in bacteria of the phyla Bacteroidetes and Firmicutes and an excess of bacteria of the phyla Actinobacteria and Proteobacteria [69]. This imbalance in the intestinal microbiome is known as dysbiosis. Li et al. showed that the expansion of fungal diversity is most likely a consequence of bacterial microbiota imbalance in CD [19]. In intensive care units, alteration of the dynamic balance between the fungal and bacterial microbiota is often observed after antibiotic or immunosuppressive therapy [70].

**Figure 4:**
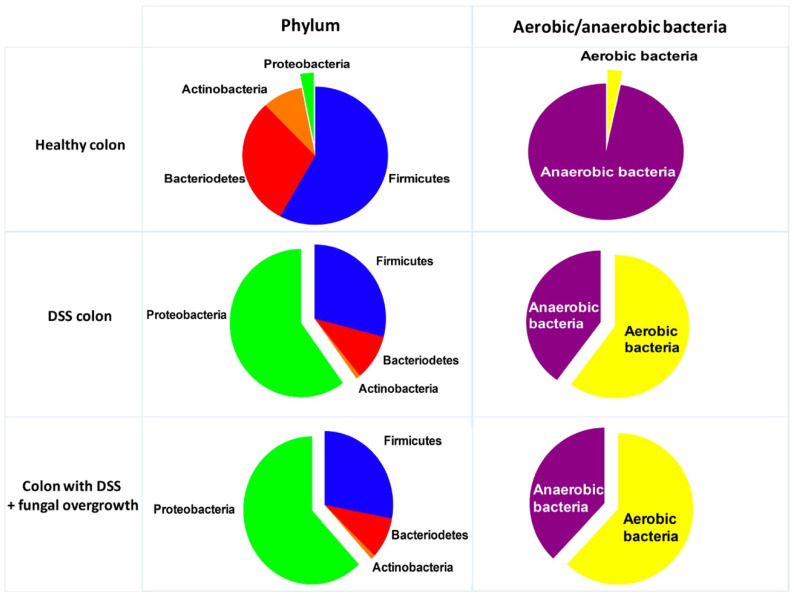
Abundance of major bacterial phyla and aerobic versus obligate anaerobes in the colons of mice with DSS-induced colitis and fungal overgrowth. Average proportions of each phylum were determined from two previous studies reporting the results in healthy mice (control), mice treated with DSS and mice with DSS and fungal overgrowth [71,72].

In the DSS-induced colitis model, an increase in Proteobacteria, mainly *Escherichia coli*, was observed during colonic inflammation and fungal overgrowth (Figure 4) [71,72]. Evidence showed that *E. coli* populations likely profit from increased oxygen availability in the inflamed colonic mucosa and exploit the inflamed gut environment to acquire a growth advantage when compared to anaerobic bacteria like Lactobacillus or Bifidobacteria [71,73]. These experimental observations are consistent with clinical studies showing that CD-associated *E. coli* with pro-inflammatory properties is adhesion-invasive *E. coli* (AIEC) [74]. AIEC increased in about 38% of patients with active CD compared to 6% in healthy subjects [74].

The gut microbiota can modulate the immune response through diverse microbial metabolites, including the production of short-chain fatty acids (SCFAs; e.g., acetate, butyrate and propionate) that exert several effects on host metabolism and the immune system [66]. SCFAs are produced from indigestible carbohydrates such as dietary fibre and resistant starch [66]. Mechanistically, SCFAs activate signalling pathways through cell surface G-protein coupled receptors (GPCRs) like GPR41 (free fatty acid receptor 3; FFAR3), GPR43 (free fatty acid receptor 2; FFAR2) and GPR109A (hydroxycarboxylic acid receptor 2; HCAR2) to induce signalling cascades that control immune functions [66,75]. In terms of the role of SCFA in antifungal activity, sodium butyrate inhibited both the growth and filamentation of *C. albicans* and enhanced the antimicrobial actions of macrophages in response to *C. albicans* sensing [76]. Metabolites secreted by some human gut-derived microbes exert antifungal activity against *C. albicans* [76,77]. Garcia et al. showed that the gut microbial metabolome has antimicrobial properties via inhibition of both *C. albicans* filamentation and fungal invasion of human colonic epithelial cells [77].

The effect of *C. glabrata* overgrowth on the gut microbiota has been also investigated in the DSS-induced colitis model [71]. This study showed an increase in populations of *E. coli*, *Enterococcus faecalis* and *Bacteroides vulgatus*, as well as a reduction in populations of anaerobes in particular, *Lactobacillus johnsonii*, *Bacteroides thetaiotaomicron* and *Bifidobacterium animalis* [71]. This bacterial reduction was more pronounced for populations of *L. johnsonii* during proliferation of *C. glabrata*. In line with this study, it has been shown that Lactobacilli antagonised *C. albicans* virulence in an in vitro gut model by shedding of *C. albicans* hyphae from the epithelial surface [78]. 

The cell wall of *C. glabrata* underwent modification during its passage through the digestive tract and showed a significant increase in cell wall chitin and β-Mans [71]. *C. glabrata* cell wall modification is more related to colonic inflammation than to bacterial dysbiosis, since this modification occurs in the days corresponding to the onset of inflammation and not while the bacterial population changes [71]. According to these findings, *C. glabrata* deficient in chitin synthase-3 caused less inflammation than the parental strain in the DSS-induced colitis model [71].

**Figure 5:**
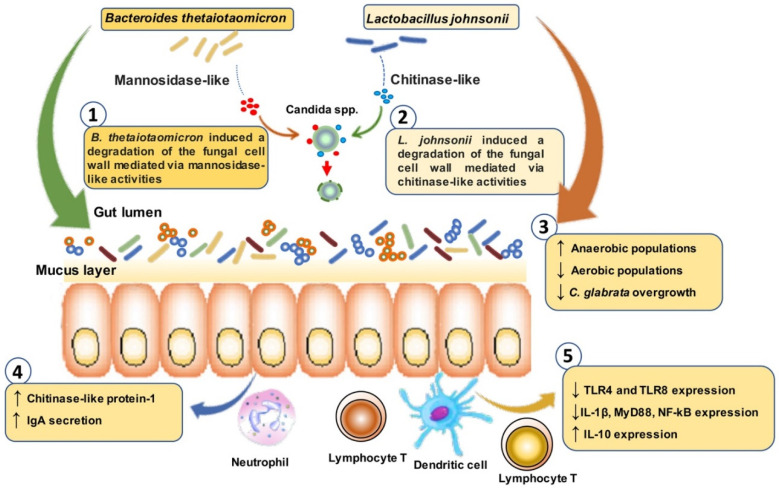
*L. johnsonii* and *B. thetaiotaomicron* decreased intestinal inflammation mediated by modulation of TLR expression and promoted the elimination of *C. glabrata* from the gut via chitinase-like and mannosidase-like activities [79].

Given that *B. thetaiotaomicron* and *L. johnsonii* populations are greatly disturbed during the development of colitis and *C. glabrata* overgrowth, oral administration of *L. johnsonii* and *B. thetaiotaomicron* restored the imbalance between aerobic and anaerobic populations of mice challenged with *C. glabrata* and treated with DSS [79]. Additionally, restoration of these two bacteria attenuated the inflammatory parameters revealed by a significant decrease in clinical and histological scores for inflammation (Figure 5). *L. johnsonii* and *B. thetaiotaomicron* also reduced the expression of pro-inflammatory mediators and enhanced anti-inflammatory cytokine responses [79]. Additionally, high chitinase-like protein-1 activation, which promotes the elimination of *C. glabrata* from the gut, was observed in these mice (Figure 5). Further investigation demonstrated that *B. thetaiotaomicron* induced degradation of *C. glabrata* α-mannan in the cell wall mediated via mannosidase-like activities while *L. johnsonii* exhibited chitinase-like activity, which was correlated with the degradation of chitin and the elimination of *C. glabrata* (Figure 5) [79].

*B. thetaiotaomicron* and *L. johnsonii* increased IgA secretion in the colon, which was correlated with a decrease in *E. coli*, *E. faecalis* and *C. glabrata* populations in mice [79]. In line with this observation, the absence of secretory IgA increased *C. albicans* hyphal growth in the mouse gut, suggesting that IgA is involved in the control of fungal commensalism in the gut [36,80,81]. Doron et al. showed that IgA produced in the gut plays a role in regulating intestinal fungal commensalism and offers a protective mechanism that might be dysregulated in CD patients [80].

## 5. Effect of Fungal Glycans on the Modulation of Intestinal Inflammation and *C. albicans* Overgrowth

**Figure 6:**
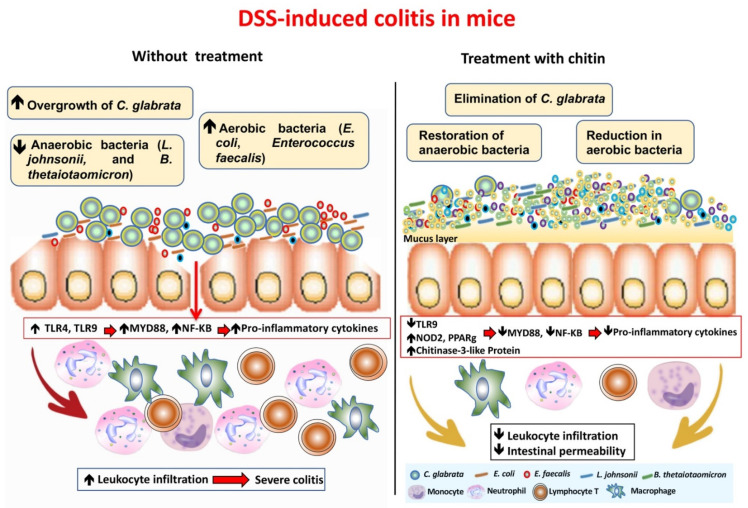
Impact of treatment with fungal chitin on the modulation of intestinal inflammation, *C. glabrata* overgrowth and the gut microbiota [71].

Chitin has been shown to have anti-ulcer, anti-tumour and anti-inflammatory activities [82,83,84]. Wagner et al. reported that oligosaccharides derived from chitin have the potential to induce IL-10 secretion through NOD-2 and TLR-9 signalling, favouring attenuation of the inflammatory response [85]. In terms of chitin digestion, chitin-degrading enzymes, known as chitinases, and chitinase-like proteins are produced by humans and other mammals [86,87]. These chitinases and chitinase-like proteins play a crucial role in the digestion of chitin-containing food and the immune defence against chitin-containing pathogens and parasites [86,87]. Evidence shows that high chitinase-3 like-protein expression was correlated with *C. glabrata* elimination from the gut (Figure 5) [71]. Additionally, oral administration of chitin in the DSS-induced colitis model reduced the number of aerobic bacteria and proliferation of *C. glabrata*, and decreased the impact of colitis mediated by the expression of TLR-8, dectin-1 and PPARγ and anti-inflammatory mediators (Figure 6) [71]. These observations are consistent with recent experimental studies, which showed that the anti-inflammatory effects of chitin are dependent on both TLR-2 and CD14 in the DSS-induced colitis model [88].

Like chitin, the molecular structure of β-glucans plays a crucial role in the immunological activities of mice and can modulate the activation or inhibition of leukocyte receptors [7]. β-Glucan has been shown to boost the host’s defence against bacterial, viral and pathogenic fungal infections [89]. Oral administration of β-glucans to mice diminished the proliferation of aerobic bacteria, in particular populations of *E. coli* and *E. faecalis*, while populations of *L. johnsonii* and *B. thetaiotaomicron* increased significantly [72]. Treatment with β-glucans increased the production of IL-10 mediated by PPARγ, promoting the attenuation of colitis and elimination of *C. glabrata* [72]. In line with this study, soluble β-glucan fractions from either *S. cerevisiae* or *C. albicans* exhibited a potent anti-inflammatory effect against colonic colitis induced by DSS in mice, indicating that oral administration of β-glucans can boost the immune response by restoring the gut microbiota and offering therapeutic perspectives for intestinal disorders and invasive fungal infections.

## 6. Role of Novel Antifungal Compounds on the Modulation of Inflammatory Parameters and Candida Overgrowth in the DSS-Induced Colitis Model

Resistance of *C. albicans* to antifungal drugs has increased considerably over the past three decades [90]. Most of the antifungal drugs available for clinical use target ergosterol, the major sterol present in fungal membranes, the biosynthesis of ergosterol or β-glucan biosynthesis [91]. The echinocandin antifungals (caspofungin, micafungin and anidulafungin) are cyclic hexapeptide agents that affect fungal cell wall biosynthesis by inhibition of β-1,3 glucan synthase [91]. Echinocandins are fungicidal against the majority of Candida spp. The fungicidal polyenes (e.g., amphotericin B) bind ergosterol in fungal cytoplasmic membranes to form membrane-spanning channels that allow leakage of essential intracellular components and fungal cell death [90,92]. The triazoles (fluconazole, itraconazole, voriconazole and posaconazole) are fungistatic antifungal drugs against Candida spp. They constitute the major class of antifungal drugs in clinical use [91]. The triazoles are heterocyclic synthetic compounds that inhibit fungal cytochrome P45014DM, which is involved in the conversion of lanosterol to ergosterol. Some *C. glabrata* strains exhibit intrinsic resistance to azoles and even susceptible strains rapidly acquire resistance, prompting clinicians to recommend echinocandin drugs as a first-line treatment for *C. glabrata* infections [93]. Currently, *C. glabrata* resistance to echinocandins is increasing and this rise is accompanied by a parallel increase in azole resistance, leading to the selection of multidrug-resistant strains [94,95]. Most alarming are the recent global outbreaks of *C. auris*, which exhibits increased resistance to all antifungal drug classes so that these antifungals are not effective therapeutic options [91,96].

Exposure of *C. albicans* to antifungal drugs triggers stress responses that allow Candida cells to benefit from different cellular responses, such as the development of mutations, gross chromosomal rearrangements, overexpression of multidrug efflux pumps and modulation of the cAMP protein kinase A (PKA) or Ca^2+^-calmodulin-calcineurin pathways [90,92]. The stress responses mediating triazole resistance activate the cyclic AMP (cAMP)-protein kinase A (PKA) signalling pathway [91]. Dumortier et al. showed that N-[2-(p-bromocinnamylamino)ethyl]-5-isoquinolinesulfonamide (H89), a PKA inhibitor, reduced the viability of *C. albicans* and decreased the expression of pro-inflammatory cytokines and innate immune receptors in colonic epithelial Caco-2 cells and macrophages [97]. Additionally, H89 decreased colonic inflammation in mice with DSS-induced colitis and allowed elimination of *C. albicans* from the gut [97]. In line with these observations, 2,3-dihydroxy-4-methoxybenzaldehyde (DHMB), a key intermediate in the synthesis of some natural compounds, including the antibacterial agents (±)-isoperbergin and perbergin, was efficient against clinically isolated caspofungin- or fluconazole-resistant *C. albicans* strains [98]. DHMB decreased the clinical and histological scores for colonic inflammation and favoured elimination of *C. albicans* from the intestine in the DSS-induced colitis model [98]. These data were corroborated by a decrease in number of aerobic bacteria, while populations of anaerobic bacteria were re-established in mice treated with DHMB [98].

In conclusion, colonic inflammation is involved in compositional changes to the mucosal bacterial microbiota and dysbiosis of the gut fungal microbiota. When the mechanisms that control the growth of these commensal organisms are disturbed, it is important to investigate whether a reduction in fungal colonisation/infection is due to: (i) gut microbial metabolites or probiotic strains such as *S. boulardii*, for which a beneficial effect has been reported on colonisation and dissemination of *C. albicans* and on intestinal inflammation; (ii) the use of fungal glycans as prebiotics, particularly chitin or β-glucans; (iii) a build-up and restoration of anaerobic bacteria, in particular *L. acidophilus* (*L. johnsonii*), *B. thetaiotaomicron* and *B. animalis*; or (iv) biosourced anti-inflammatory/anti-fungal compounds. These strategies may lead to the development of new and exciting ways to modulate colonic inflammation and control fungal overgrowth.

## Data Availability

Not applicable.
